# Experimental Study on Acoustic Emission Signals Under Different Processing States of Laser-Assisted Machining of SiC Ceramics

**DOI:** 10.3390/mi17010042

**Published:** 2025-12-29

**Authors:** Chen Cao, Yugang Zhao, Xiukun Hu, Xiao Cui

**Affiliations:** 1 School of Mechanical Engineering, Taishan University, Taian 271000, China; 2 Key Laboratory for Advanced Forming Technology and Equipment, Key Laboratory of High-Efficiency and Clean Mechanical Manufacture (Ministry of Education), School of Mechanical Engineering, Shandong University, Jinan 250061, China; 3 School of Mechanical Engineering, Shandong University of Technology, Zibo 255049, China

**Keywords:** laser-assisted machining (LAM), SiC ceramics, surface morphology, acoustic emission (AE), processing state

## Abstract

In this paper, laser-assisted machining (LAM) of SiC ceramics was taken as the research object, and the different spectrum and energy spectrum characteristics and their changing trends of acoustic emission (AE) signals under processing states of brittleness, plasticity and thermal damage were analyzed. The numerical characterization of ceramic softening degree was indirectly realized by the energy spectrum characteristics of low-frequency band energy ratio, marking a methodological breakthrough in transitioning from qualitative analysis to quantitative detection for identifying plastic processing state. First, the surface morphology of the machined surface based on the single-factor experiment of laser power was analyzed, and three different processing states and ranges of laser power were determined, namely brittle state (0–185 W), plastic state (185–225 W) and thermal damage state (>225 W). Then, the wavelet packet denoising and spectrum analysis of AE signals under different processing states were carried out to obtain the corresponding frequency of the maximum amplitude and the amplitude change trend of the characteristic frequency (515 kHz) in the high-frequency domain. Finally, the energy spectrum analysis of acoustic emission signals was carried out, and the method of indirect characterization of ceramic softening degree by low-frequency band energy ratio was proposed. This paper provides a numerical characterization method and theoretical guidance for the detection and identification of the plastic processing state of ceramic laser-assisted cutting.

## 1. Introduction

The ongoing evolution of science and technology necessitates enhanced support for high-performance components, driving a rapidly expanding market demand for engineering ceramic parts [1]. Engineering ceramics possess a range of advantageous properties, such as exceptional hardness, outstanding wear resistance, and superior thermal stability, which render them highly suitable for operation under demanding conditions, including extreme temperatures and corrosive environments [2,3]. Due to their excellent physical and chemical properties, they are widely used in aerospace, bioengineering, medicine, the nuclear industry, automotive industry and other fields [4,5,6]. However, the high hardness, high strength and high brittleness of ceramic materials are not only causes of low processing efficiency, but also easily lead to surface damage in the processing process [7,8,9]. The significant market demand imposes higher requirements on the processing technology for engineering ceramics. The rapid development of laser technology has become the premise of LAM technology, and high-energy laser beams have become one of the most effective processing tools. LAM employs a high-energy laser beam to preheat the surface of the workpiece, thereby inducing localized softening of specific areas. This process effectively reduces the hardness and yield strength of the material within the softened regions, facilitating subsequent machining operations by minimizing resistance and enhancing material formability. During cutting, the deformation behavior of the material changes from brittleness to plasticity, reducing or eliminating crack and pit defects on the surface, not only improving the processing efficiency, but also obtaining good surface quality [10,11]. The softening degree of the processing area of the ceramic workpiece is the key to realizing the continuous and stable plastic processing state of LAM.

At present, in order to evaluate the softening degree of materials during processing, the measured temperature of the heating position or a fixed reference position relative to the heating position is used as the basis for evaluating the softening degree of materials in the research literature [12,13,14,15].The surface temperature can facilitate only the theoretical fuzzy judgment of the softening degree of the workpiece, and the heating and softening during the actual processing are the processes of heat absorption and a volume effect. Even if the surface temperature reaches the softening temperature, the tool machining part inside the material will not be softened, and the plastic cutting of LAM cannot be realized. Therefore, despite the great efforts of researchers, it is still not possible to achieve a reliable numerical characterization of the softening degree by surface temperature. Therefore, this paper focuses on LAM of SiC ceramics and proposes an indirect method to characterize softening degree based on the frequency band energy ratio of AE signals.

AE is a phenomenon of the strain energy inside the material that is released in the form of transient elastic waves when the material undergoes deformation or fracture [16,17]. Mechanical, thermal, and chemical stresses can all cause the release of strain energy [18]. AE is extremely sensitive to changes in the microstructure of materials, including plastic deformation, fracture, crack initiation and propagation, phase transitions, corrosion, and other forms of failure. Therefore, AE is widely used as a means of nondestructive testing in the monitoring of machining processes. Cho and Komvopoulos employed acoustic emission (AE) to monitor the machining process and tool condition during turning of AISI 4340 steel using multilayer-coated tools. Their findings indicated that continuous signals were low-frequency, while burst signals were high-frequency. The low-frequency signals arose from plastic deformation occurring in the primary, secondary, and tertiary shear zones, as well as in the near-surface tool regions, and were strongly influenced by contact at the tool/workpiece and tool/chip interfaces. High-frequency signals originated from cracks on the tool surface [19]. Prakash et al. examined the impact of tool wear on chip formation mechanisms and chip morphology during milling of aluminum alloy by AE. The results showed that chip breakage and tool edge chipping produced burst signals with high amplitude and high frequency. The primary, secondary and tertiary deformation zones produced continuous signals with low amplitude and low frequency [20]. Tangjitsitcharoen and Lohasiriwat proposed a numerical control wear detection system for turning tools based on wavelet transform. The signal of chip breakage belonged to the high-frequency signal, while the tool wear signal belonged to the low-frequency signal, which provided a reasonable basis for the extraction of tool wear signal characteristics [21].

In view of the previous literature research, this paper analyzed the surface morphology of LAM of SiC ceramics to determine the brittleness, plasticity and thermal damage processing state. Then, the wavelet packet denoising and spectrum analysis were applied to the acoustic emission signals of different processing states, and the corresponding frequency of the maximum amplitude and the amplitude variation trend of the characteristic frequency (515 kHz) in the high-frequency domain were obtained. Finally, the acoustic emission signal spectrum analysis was carried out to propose an indirect method to characterize the softening degree of ceramic material by the energy ratio of the low-frequency band.

In addition to the above, this article also includes the following four sections. Section 2 presents the experimental materials and equipment, detailing the material performance parameters and the functional specifications of each equipment component. Section 3 not only introduces the research basis involved in the process of LAM of SiC ceramics, including the source of AE signal and the analysis of softening degree characterization method and signal feature extraction, but also introduces the experimental scheme. Section 4 presents the experimental results, and Section 5 provides a summary of the entire document.

## 2. Experimental Materials and Equipment

The external dimensional characteristics of experimental samples fabricated from pressureless sintered silicon carbide (SiC) ceramics are Φ11 × 45 mm^2^, and its key material property parameters are shown in Table 1.

**Figure 1:**
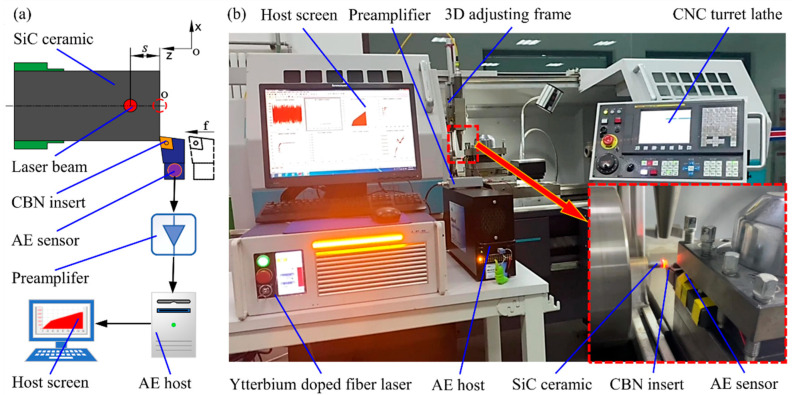
Experimental platform: (**a**) Schematic diagram; (**b**) Actual layout.

Figure 1 shows the schematic diagram and the actual layout of the experimental equipment. The experimental equipment consists of two parts: CNC machine tool with LAM function and signal processing system. The CNC machine tool with LAM function includes the original CNC turret lathe (CKD6136i, Dalian Machine Tools Group, Dalian, China), the three-dimensional adjustment frame and the ytterbium-doped laser (YLR-150/1500-QCW-MM-AC, IPG Photonics Co., Oxford, MA, USA). After the continuous laser emitted by the ytterbium-doped fiber laser propagates through the fiber, the high-energy laser beam with a maximum power of 250 W vertically irradiates the surface of the workpiece. The three-dimensional adjustment frame is constructed from aluminum profile components that can realize laser adjustment in three directions of X, Y and Z axes. In addition, a CBN insert (CNGA120408FBS7000) and a supporting toolholder (MCLN2020K12) are selected for LAM of SiC ceramics. During the turning process, the laser beam and the tool move synchronously at the feed speed *f* from the starting position (dotted line). After the preheating distance *S* = 1 mm, the tool tip contacts the workpiece. The acoustic emission sensor (Nano30, Physical Acoustics Corporation, Princeton Junction, NJ, USA) and the tool holder are filled with butter coupling agent and then fixed with tape. The signal is transmitted through the AE sensor and preamplifier (gain of 20 dB) to a signal acquisition system and displayed on the screen.

Furthermore, the micromorphology of the machined surface is analyzed using 3D digital microscope (DSX1000, Olympus, Tokyo, Japan) and SEM (Quanta, FEI, Fremont, CA, USA).

## 3. Research Fundamentals and Experimental Methods

### 3.1. Sources of AE Signals

**Figure 2:**
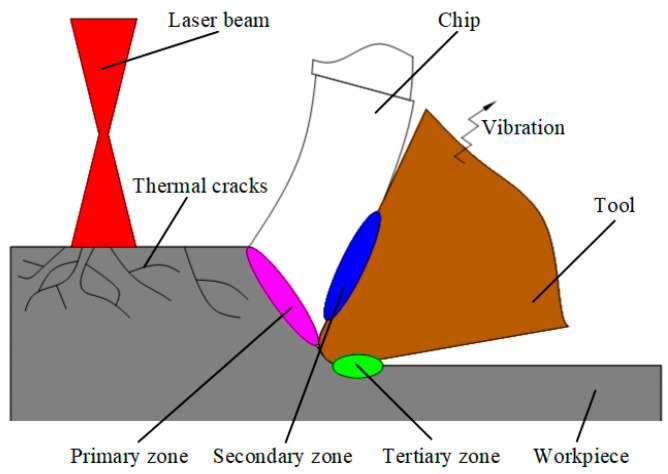
AE sources from LAM.

AE signals mainly come from microstructural changes in deformed materials, such as point defects and dislocation motion, twinning and slippage of grain boundaries in polycrystalline materials, plastic deformation, void generation and collapse, inclusion breakage, phase transition, crack initiation and growth, and fracture [19]. The AE signal in the processing process can be divided into a continuous signal and burst signal. The main sources of continuous signals are plastic deformation and tool wear, the signal amplitude is relatively low, and the burst signal is mainly derived from chip breakage, built-up edge generation and removal, tool fracture and chip curling, and the signal amplitude is relatively high [22,23]. Figure 2 shows that in addition to the AE sources in the conventional cutting process, the AE sources generated by laser preheating are also included in LAM.

During laser preheating of SiC ceramics, due to the different grain orientation and different thermal expansion coefficients within the material, stress concentration occurs at the grain boundaries, and the resulting thermal stress leads to the initiation and expansion of cracks on the preheated surface of the workpiece. In the laser-assisted turning of SiC ceramics, a large number of grain boundary slip avalanches occur in the workpiece material caused by dislocation motion in the primary shear zone, eventually generating a large amount of plastic flow and chip formation, in the secondary shear zone (tool/chip interface) and tertiary shear zone (tool/workpiece interface), both generating a large number of shear flows and sliding. The specific sources of continuous and burst signals are shown in Table 2.

### 3.2. Analysis of Characterization Methods for Softening Degree and Signal Feature Extraction

From the analysis of the source of AE signals, it can be concluded that the AE signals generated in LAM can also be divided into low-frequency continuous signals and high-frequency burst signals by signal processing. Ravindra et al. found that the softening effect on SiC ceramic material gradually increased with the increase in heating temperature, and its hardness and yield strength gradually decreased. The yield strength at 1500 °C was significantly lower than its fracture strength [24]. In addition, the temperature distribution and the depth of the softening layer have been thoroughly studied in our published paper [25]. In LAM of SiC ceramics, the softening degree of the material on the whole is gradually increased by continuous laser preheating, the plastic deformation ratio of the material in the deformation zone is gradually increased during the cutting process, and the brittle fracture ratio is decreased. The corresponding low-frequency band energy ratio of the continuous AE signal increases, and the high-frequency band energy ratio of the burst AE signal decreases. Therefore, the ratio of the low-frequency characteristic band energy of the continuous AE signal is selected as the characteristic value of the softening degree.

In addition, the AE signal has the characteristics of non-linearity and non-stationarity. The Fourier transform cannot reveal the instantaneous structure of the signal, and cannot determine the variation in the frequency component with time. It is only suitable for the analysis of stationary signals. The discrete wavelet transform is proposed to solve the problem of non-stationary signal processing. Although the discrete wavelet transform has flexible time-frequency resolution, its high-frequency resolution is relatively low. The wavelet packet transform further improves the discrete wavelet transform, which not only retains the resolution of the low-frequency domain, but also obtains the high resolution of the high-frequency domain. Therefore, the wavelet packet transform is selected as the analysis method for processing AE signals.

**Figure 3:**
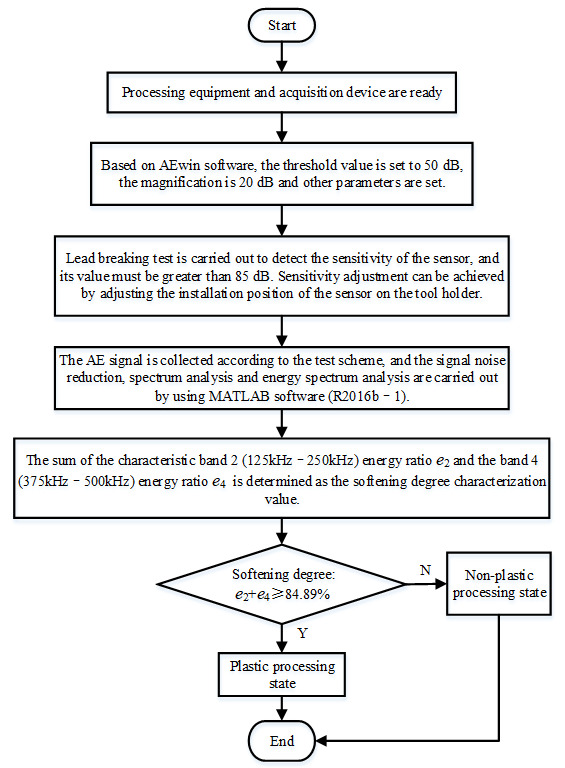
Signal feature extraction and processing state identification.

In order to realize the feature extraction of the AE frequency domain signal, in addition to the reliable operation of the processing equipment, the sensitivity of the signal acquisition sensor must meet the requirements for installation and use. The process of AE signal feature extraction is shown in Figure 3.

### 3.3. Experimental Scheme Design

Compared with the traditional turning method, the preheating effect of the laser in LAM is a volumetric effect, which significantly reduces the hardness and yield strength of SiC ceramics and achieves the softening of the material. The laser is the energy source that directly affects the softening degree of ceramic material, so the laser power is selected for the single-factor experiments.

Single-factor experiments are carried out to investigate the effects of laser power on the softening degree. The values for each factor under standard experimental conditions are as follows: laser power 205 W, rotational speed 1620 r/min, feed speed 3 mm/min and cutting depth 0.15 mm. The range of values of the parameters of the single factor is shown in Table 3. Among them, 0 W represents the conventional cutting conditions, and no laser heating is involved.

## 4. Results and Discussion

### 4.1. Analysis of Machined Surface Morphology and Processing State

**Figure 4:**
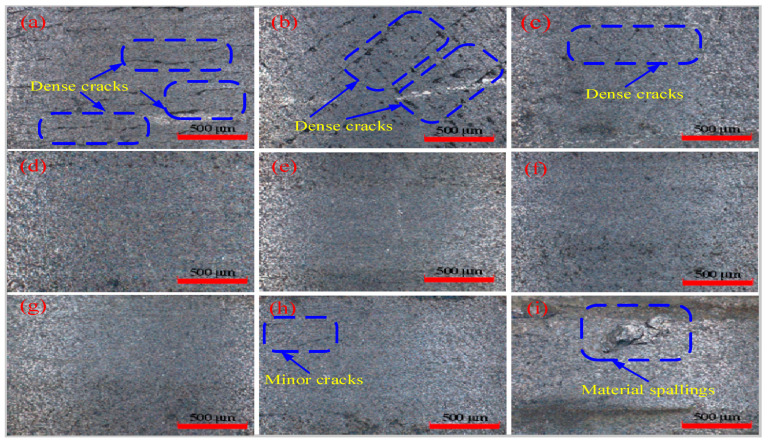
Machined surface morphology (3D digital microscope): (**a**) 0 W; (**b**) 150 W; (**c**) 175 W; (**d**) 185 W; (**e**) 195 W; (**f**) 205 W; (**g**) 215 W; (**h**) 225 W; (**i**) 230 W.

Figure 4 presents the machined surface morphology under varying laser powers, as observed via 3D digital microscopy in our own previous literature [26]. Figure 4a shows that a large number of dense cracks are evenly distributed on the machined surface in traditional turning without laser preheating. Figure 4b indicates that the machined surface preheated with a 150 W laser exhibits large, elongated strip-like cracks accompanied by tearing defects; however, the defect density remains relatively low. It can be concluded that the material softens, the yield strength gradually decreases, the brittle removal ratio of the material decreases, and the plastic removal ratio increases. Figure 4c demonstrates that the surface morphology of the sample subjected to 175 W laser preheating has been significantly enhanced, with a marked reduction in crack formation. Only in some areas (blue marked areas), there are small dense cracks. It can be inferred that the degree of plastic removal of the material is improved. Figure 4d shows that the machined surface preheated by 185 W laser has no cracks or other defects, and the surface morphology is good. Figure 4e–g demonstrate that the machined surface exhibits no cracks or other defects, with excellent uniformity and significantly enhanced surface quality. It can be inferred that as laser power increases, the extent of material softening improves further, leading to a higher degree of plastic removal. Figure 4h shows the machined surface morphology obtained with 225 W preheating. The overall surface finish is of good quality, and only a few tiny cracks appear in the very small areas (blue marked areas). Figure 4i indicates that the machined surface preheated at 230 W has serious defects, and the excessive laser power leads to high thermal stresses that exceed the critical value of the material, causing the initiation and propagation of surface cracks and ultimately resulting in material spalling.

Based on the aforementioned analysis, the following conclusions regarding the processing states of the machined surfaces in Figure 4 can be drawn: The surface in Figure 4a exhibits a brittle processing state. The surfaces in Figure 4b,c transition from a brittle to a plastic processing state. The surfaces in Figure 4d–h are in a plastic processing state. The surface in Figure 4i demonstrates a thermally damaged processing state.

**Figure 5:**
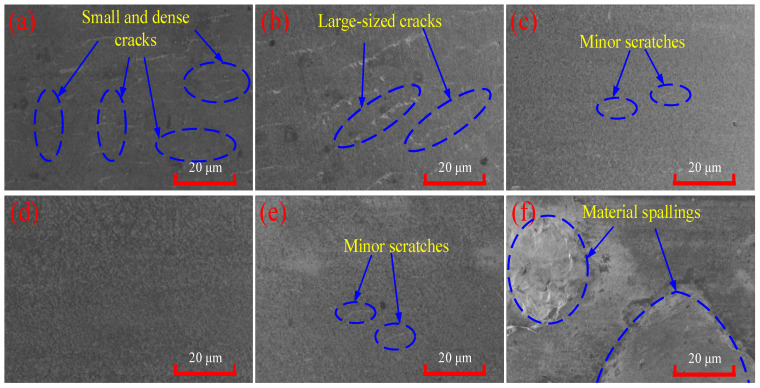
Machined surface morphology (SEM): (**a**) 0 W; (**b**) 150 W; (**c**) 185 W; (**d**) 215 W; (**e**) 225 W; (**f**) 230 W.

To further verify the analysis results of Figure 4, the surface morphology at the key laser power is analyzed by SEM [26]. Figure 5a indicates that the traditional turning surface without laser preheating is also widely distributed with small and dense microcracks. Figure 5b shows that there are large-sized and loosely distributed cracks on the machined surface at 150 W preheating, accompanied by slight tearing defects. Figure 5c indicates that the machined surface at 185 W preheating exhibits good morphology and uniformity, with only a small blue-marked area showing slight cracks or scratches. Figure 5d demonstrates excellent machined surface morphology at 215 W preheating; the entire area is free of cracks, pits, and other defects, resulting in superior surface quality. Figure 5e demonstrates that the machined surface morphology at 225 W preheating is marginally inferior to that in Figure 5d but remains excellent, with only minor cracks or scratches in a small blue-marked area. Figure 5f reveals very poor surface morphology at 230 W preheating, characterized by severe material spalling due to thermal stress.

Based on the surface morphology analysis, three distinct processing states of the machined surface correspond to specific laser power ranges: brittle state (0–185 W), plastic state (185–225 W), and thermal damage state (>225 W). The critical transition points are 185 W, marking the brittle-to-plastic transition, and 225 W, indicating the plastic-to-thermal damage transition.

### 4.2. AE Signal Processing and Analysis

#### 4.2.1. Signal Denoising

According to the advantages of the wavelet packet transform described above, different wavelet basis functions are selected to denoise the AE signal. The noise reduction effect is evaluated by three calculation Equations (1)–(3), namely signal-to-noise ratio (*SNR*), mean square error (*MSE*) and root mean square error (*RMSE_S_*). The larger *SNR* and the smaller *MSE* and *RMSE_S_* represent the better signal noise reduction effect. In this paper, five wavelet basis functions of haar, coif2, db6, sym6 and sym8 are selected to perform five-layer decomposition of the signal. The fixed global threshold is set to 0.15 in the denoising process. The sampling frequency is 2 MSPS, the recording length is 4K, and the analog filter range is 100 kHz–3 MHz. The 0.5 s AE signal collected under standard experimental conditions is selected as a sample to compare the denoising effect. Table 4 shows the denoising results of different wavelet basis functions.

(1) $$SNR=10\mathrm{lg}\frac{\sum f{\left(t\right)}^{2}}{\sum {\left[f(t)-f(t{)}^{\prime }\right]}^{2}}$$

(2) $$MSE=\frac{{\sum \left[f(t)-f(t{)}^{\prime }\right]}^{2}}{n}$$

(3) $$RMSE_{S}=\sqrt{\frac{\sum {\left[f(t)-f(t{)}^{\prime }\right]}^{2}}{n}}$$

where $f\left(t\right)$ is the original signal, $f\left(t\right)$′ is the signal after denoising, and *n* is the number of sampling points.

Table 4 shows that the maximum *SNR* of the sym8 wavelet basis function is 21.033, the minimum *MSE* is 0.0168, and the minimum *RMSE_S_* is 0.1298. Therefore, the sym8 wavelet basis function has the best denoising effect.

#### 4.2.2. Frequency Spectrum Analysis

**Figure 6:**
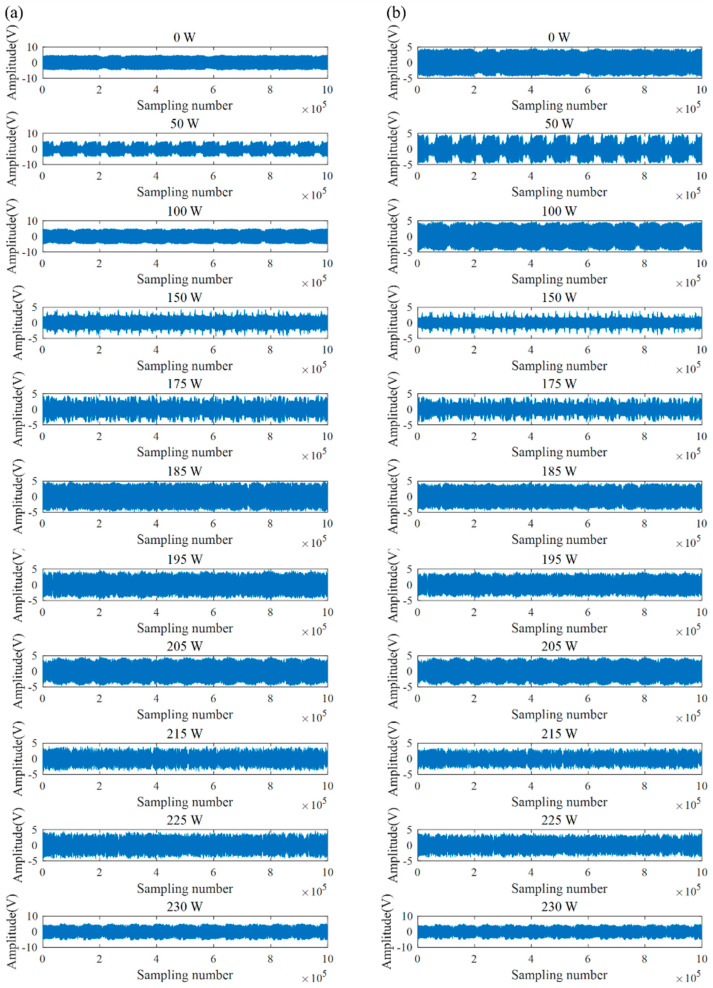
Signal denoising with different laser powers: (**a**) Original signal; (**b**) Denoised signal.

Before frequency spectrum analysis, the sym8 wavelet basis function is used to denoise the 0.5 s AE signal collected based on laser power single-factor experiments. Figure 6 shows the original signal and the denoised signal of different laser powers.

**Figure 7:**
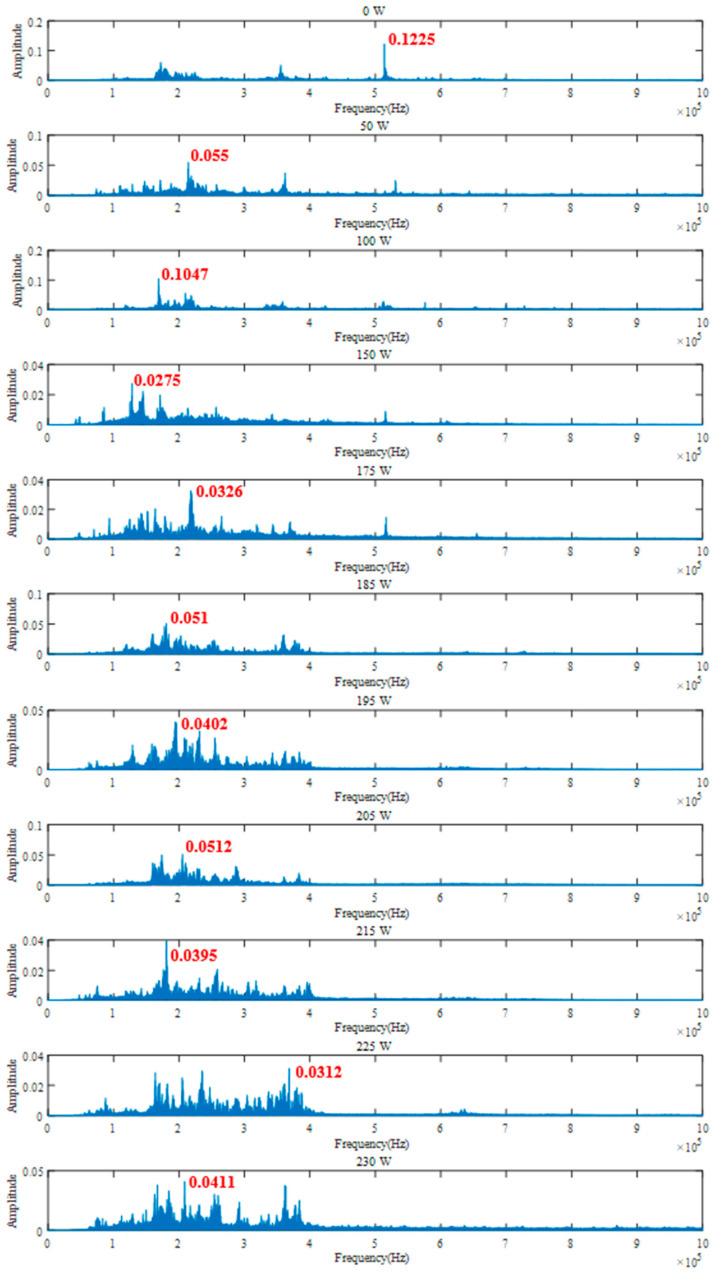
Signal frequency spectrum under different laser powers.

To further study the frequency domain characteristics of AE signals, fast Fourier transform (FFT) is performed on the denoised signal. Figure 7 is the frequency spectrum of AE signals with different laser powers. Through comparative analysis, it can be found that the signal components are mainly concentrated in the low-frequency domain of 0–500 kHz. Although the high-frequency domain signal components of 500–1000 kHz are less, they also contain important information related to the processing state. When the laser power is 0 W (without laser preheating), the maximum amplitude appears at 513.8 kHz in the high-frequency domain, and the amplitude is 0.1225. As the laser power gradually increases, not only does the maximum amplitude of the signal gradually move to the low-frequency domain, but also the signal amplitude of the frequency near 515 kHz decreases overall.

**Figure 8:**
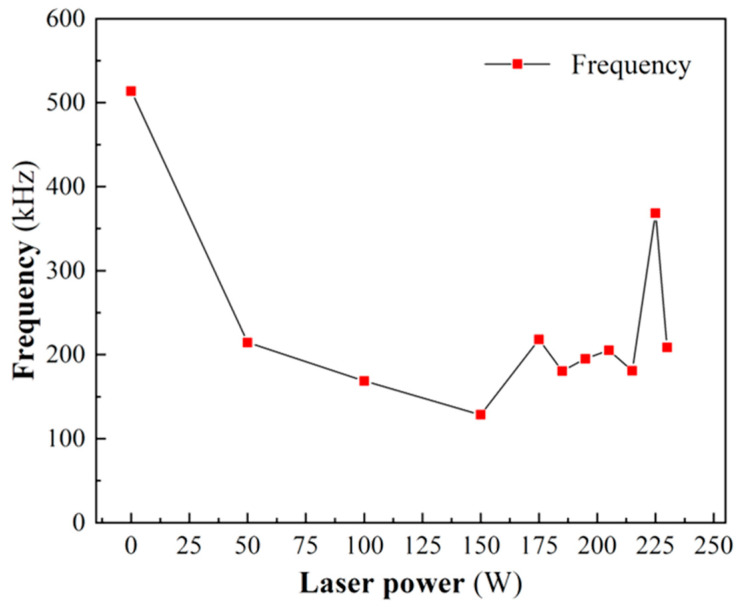
Maximum amplitude corresponding to frequency of signal under different laser powers.

Table 5 and Figure 8 show the corresponding frequencies of the maximum amplitude of the signal under different laser powers. Figure 8 shows that as the laser power increases, the frequency of the maximum amplitude of the signal gradually shifts from the high-frequency domain to the low-frequency domain, showing the trend of sharp decrease at first, stabilization in the middle, and finally a small increase. In the brittle processing state (0–185 W), as the laser power increases, the ceramic softening degree gradually increases, and the frequency decreases from 513.8 kHz at 0 W to the minimum value of 128.4 kHz at 150 W. When the laser power exceeds 175 W, the material surface begins to harden due to thermal stress, generating thermal cracks and gradually increasing [25], resulting in a slight increase in frequency. In the plastic processing state (185–225 W), the number and strength of cracks are relatively stable, and although the frequency fluctuates, it is still relatively stable. In the thermal damage processing state (>225 W), the degree of hardening of the material increases, the degree of softening decreases, and the number of thermal cracks increases, resulting in a slight increase in the signal frequency, reaching 368.4 kHz, but still in the low-frequency domain.

**Figure 9:**
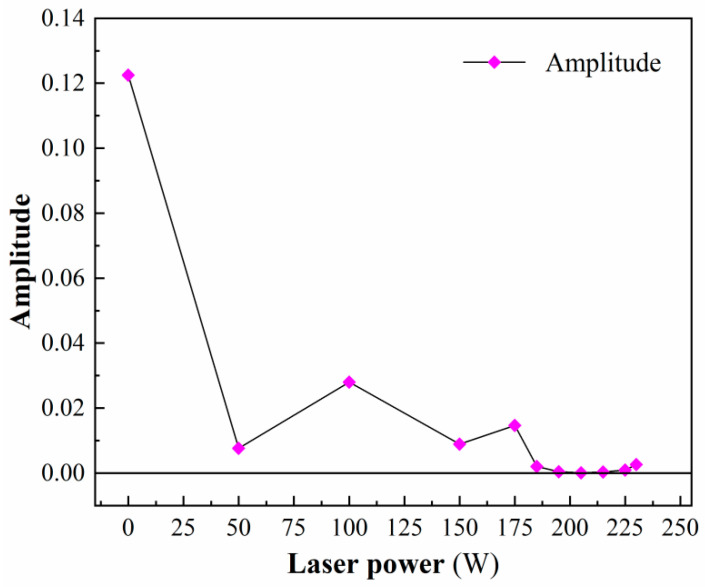
Amplitude of frequencies near 515 kHz.

Table 6 and Figure 9 show the amplitude of the signal at frequencies around 515 kHz under different laser powers. Figure 6 shows that as the laser power increases, the amplitude of the frequency near 515 kHz decreases significantly overall. In the brittle processing state (0–185 W), laser preheating has a significant softening effect on the surface of the workpiece, with a rapid increase in the proportion of low-frequency signals, resulting in a significant decrease in the amplitude of high-frequency signals. The amplitude decreases sharply from 0.1225 at 0 W to 0.0076 at 50 W, and then stabilizes. In the plastic processing state (185–225 W), as the softening degree of the material continues to increase, the proportion of low-frequency signals is higher and dominant. The maximum amplitude of the high-frequency signals is 0.002, and the minimum amplitude is 0.0001, which is significantly smaller than the amplitude of the brittle processing state. In the thermal damage processing state (>225 W), although low-frequency signals still dominate, the thermal cracks caused by laser preheating increase the high-frequency component of the signal, resulting in a slight increase in the amplitude of the high-frequency domain. Therefore, the maximum amplitude in the high-frequency domain is 0.0026, which shows a slight increase compared to the plastic processing state.

#### 4.2.3. Energy Spectrum Analysis

In order to further investigate the signal characteristics, energy spectrum analysis is conducted on denoised signals with different laser powers. Based on Shannon theory, the wavelet packet analysis method is used to decompose the denoised signal into three layers using the sym8 wavelet basis function. The high-frequency and low-frequency components of the signal are separated to obtain eight sub-bands with a bandwidth of 125 kHz. The energy analysis of each band is used to identify the frequency domain characteristics of the signal, determine the characterization parameters of softening degree, and ultimately achieve the recognition of processing state. The energy and energy ratio of the signal sub-band satisfy Equations (4) and (5):

(4) *E* = *E*_1_ + *E*_2_ + *E*_3_ + *E*_4_ + *E*_5_ + *E*_6_ + *E*_7_ + *E*_8_

(5) 1 = *e*_1_ + *e*_2_ + *e*_3_ + *e*_4_ + *e*_5_ + *e*_6_ + *e*_7_ + *e*_8_

where the total energy of the signal is denoted as *E*, the energy of each sub-band (from low frequency to high frequency) is denoted as *E*_1_–*E*_8_, and the energy ratio is denoted as *e*_1_–*e*_8_, respectively.

**Figure 10:**
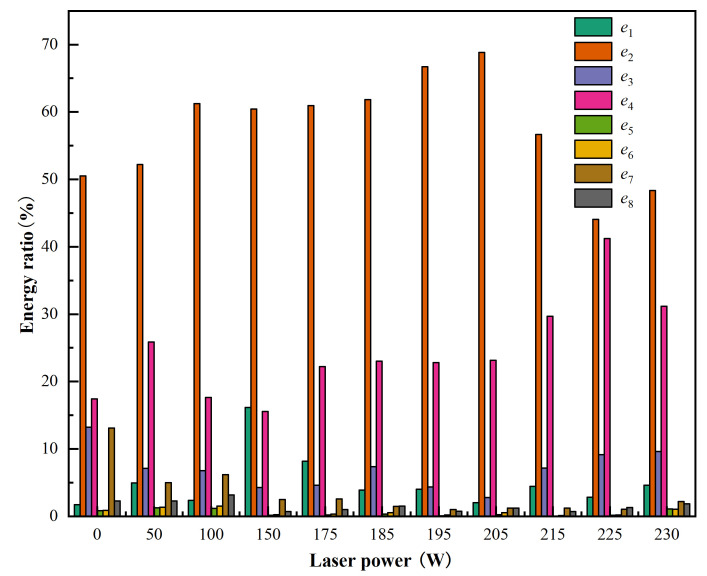
Frequency band energy ratio under different laser powers.

Table 7 and Figure 10 show the energy ratio of sub-bands under different laser powers. The comparison shows that the signal energy is mainly concentrated in the first four low-frequency bands (0–500 kHz), especially band 2 (125–250 kHz) and band 4 (375–500 kHz), and the remaining energy is distributed in the last four high-frequency bands (500–1000 kHz), especially band 7 (750–875 kHz).

**Figure 11:**
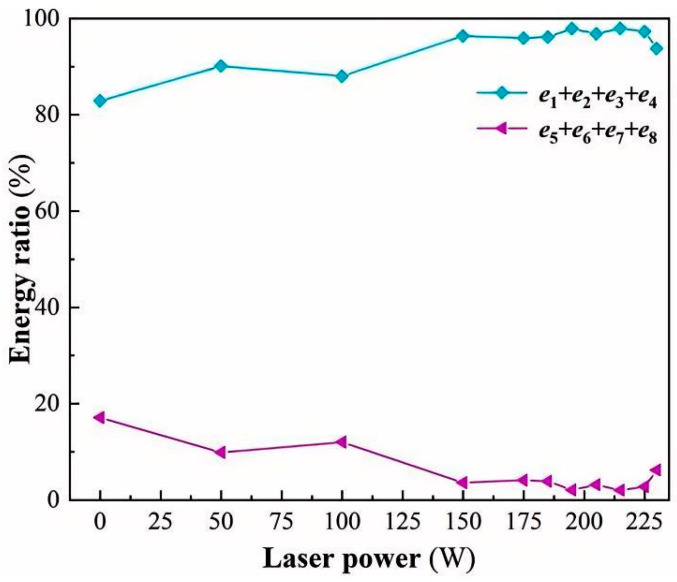
Energy ratio of low-frequency and high-frequency bands.

To investigate the relationship between the correlation band energy ratio and the softening degree, the trend of the correlation band energy ratio with the laser power is further analyzed. Figure 11 indicates that as the laser power increases, the energy ratio of the low-frequency band (0–500 kHz) gradually increases from 82.89% at 0 W to 97.96% at 215 W, and then gradually decreases to 93.77% at 230 W. On the contrary, Figure 11 shows that as the laser power continues to increase, the energy ratio of the high-frequency band (500–1000 kHz) gradually decreases from 17.11% at 0 W to a minimum of 2.04% at 215 W, and then rises to 6.23% at 230 W. It can thus be inferred that with increasing laser power, the material softening effect initially strengthens during the brittle processing stage, accompanied by a progressive rise in the low-frequency band energy ratio and an enhancement in softening degree. Upon entering the plastic processing stage, both the low-frequency band energy ratio and the softening degree reach their maximum values. However, when the process transitions into the thermal damage stage, the softening effect diminishes, the low-frequency band energy ratio declines, and the degree of softening follows an inverse trend.

#### 4.2.4. Softening Degree Characterization and Processing State Identification

Based on an analysis of the frequency and energy spectra, the following correlations with increasing laser power are observed: The frequency corresponding to the maximum amplitude initially decreases significantly, then stabilizes, and finally shows a slight increase, corresponding sequentially to the brittle, plastic, and thermal damage processing states. Concurrently, the amplitude at the characteristic frequency (515 kHz) exhibits a marked decline, followed by a plateau, and then a minor rise in the high-power regime. Furthermore, the energy ratio within the low-frequency band (0–500 kHz) progressively increases, stabilizes, and subsequently experiences a slight decrease, again mapping onto the brittle, plastic, and thermal damage states, respectively.

**Figure 12:**
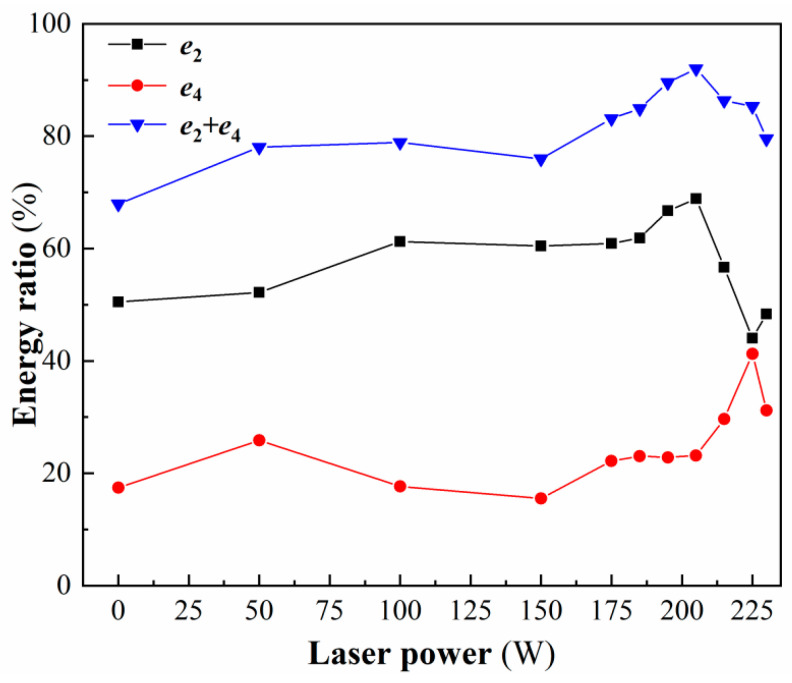
Energy ratio of band 2, band 4 and the sum of the two.

The change in the softening degree directly leads to changes in the frequency spectrum and energy spectrum, and the trend of the softening degree is consistent with the trend of the energy ratio in the low-frequency band (0–500 kHz). Therefore, frequency bands 2 (125–250 kHz) and 4 (375–500 kHz), which are highly correlated in the lower frequency bands, are selected for characterization of the softening degree and identification of processing states. Figure 12 presents the energy ratios of frequency bands 2 and 4, along with their combined sum, as a function of laser power. As the power rises from 0 W to 150 W, the increasing material softening promotes a greater proportion of plastic removal during chip formation, without thermal cracking on the preheated surface. This leads to a rise in the energy ratio of band 2 and a corresponding decrease in band 4. When the power reaches 205 W, softening continues to intensify, and plastic removal further increases. Although surface corrosion and thermal crack initiation begin at 175 W, their frequencies fall outside band 4. Consequently, the energy ratio of band 2 increases further to its maximum, while that of band 4 remains stable. When the laser power reaches 225 W, heightened thermal stress accelerates crack propagation, resulting in an increased energy ratio in band 4 and a decline in band 2. Upon reaching 230 W, greater thermal stress causes surface material spallation, whose frequency exceeds the range of band 4, leading to a decrease in this band’s energy ratio. Meanwhile, ongoing plastic material removal during cutting produces a slight renewed increase in the energy ratio of band 2.

The variation trend of the sum of the energy ratio of frequency band 2 and frequency band 4 is consistent with the variation trend of the energy ratio of the low-frequency band (0–500 kHz). Additionally, the sum values in different processing states are quite different, and the resolution is stronger. Therefore, the sum of the energy ratio of frequency band 2 and frequency band 4 is defined as the softening degree, i.e., *e*_2_ + *e*_4_. In the plastic processing state, the minimum value of softening degree is 84.89% (185 W), and the maximum value is 92.01% (205 W). Therefore, when the softening degree is not less than 84.89%, the cutting process is in a plastic state; when the softening degree is less than 84.89%, the cutting process is in a non-plastic state.

## 5. Conclusions

In this paper, laser-assisted machining (LAM) of SiC ceramics is taken as the research object, and the different spectrum and energy spectrum characteristics and their changing trends of acoustic emission (AE) signals under three processing states of brittleness, plasticity and thermal damage are analyzed. The numerical characterization of ceramic softening degree is indirectly realized by the energy spectrum characteristics of the low-frequency band energy ratio. The specific contents are summarized as follows:

1. Single-factor experiments on laser power were conducted to analyze the machined surface morphology of SiC ceramics using 3D digital microscopy and SEM. Results indicate that three distinct processing states correspond to specific laser power ranges: brittle state (0–185 W), plastic state (185–225 W), and thermal damage state (>225 W). The critical transition points between brittle and ductile behavior occur at 185 W, while the transition from plastic to thermal damage occurs at 225 W.
2. Based on single-factor laser power experiments, the collected AE signals were denoised using the sym8 wavelet basis function, followed by frequency spectrum and energy spectrum analysis. The frequency spectrum analysis reveals that as the laser power increases, the frequency corresponding to the maximum amplitude initially decreases significantly, then stabilizes, and finally exhibits a slight increase. This trend corresponds respectively to the brittle, plastic, and thermal damage processing states. A similar pattern is observed for the amplitude at the characteristic frequency of 515 kHz in the high-frequency domain. The energy spectrum analysis indicates that the energy ratio of the low-frequency band (0–500 kHz) first rises gradually, remains stable, and then decreases slightly with increasing laser power, again corresponding to the brittle, plastic, and thermal damage states.
3. This paper proposes a characterization method for softening degree, and the plastic processing state of the materials can be identified by the softening degree. The softening degree is defined as the sum of the energy ratios of the characteristic frequency band 2 (125–250 kHz) and frequency band 4 (375–500 kHz) in the low-frequency band. When the softening degree is not less than 84.89%, the cutting process is in a plastic state; when the softening degree is less than 84.89%, the cutting process is in a non-plastic state.

## Figures and Tables

**Table 1:** Key material property parameters.

Items	SiC
Elastic modulus (GPa)	290
Vickers hardness (kgf·mm^−2^)	2100
Compressive strength (MPa)	3000
Fracture toughness (MPa·m^1/2^)	4
Thermal expansion coeff. × 10^−6^/℃	4.5
Thermal conductivity (W/mK)	80
Melting point (K)	3100
Specific heat capacity (J/kgK)	1100
Density (g/cm^3^)	3.15

Note: Parameters supplied by Guangdong XY Fine Ceramic Technology Co., Ltd. (Dongguan, China).

**Table 2:** Types and sources of AE signals.

Signal Types	Sources
Continuous AE signals (low-frequency)	Formation process (shear process) of chips
	Rubbing between the cutting tool and chips or the workpiece
	Wear of the cutting tool
Transient AE signals (high-frequency)	Thermal cracks due to thermal stresses caused by laser preheating
	Collisions and breakages of chips
	Formation and removal of built-up edges
	Damage to the cutting tool (breakage, chipping, flaking, etc.)
	Entanglement of chips onto the workpiece or cutting tool
	Vibrations of the cutting tool

**Table 3:** Experimental parameters.

No.	Process Parameter	Value Range
1	Laser power (W)	0, 50, 100, 150, 175, 185, 195, 205, 215, 225
2	Rotational speed (r/min)	1620
3	Feed speed (mm/min)	3
4	Cutting depth (mm)	0.15

**Table 4:** Results of signal denoising.

Items	haar	coif2	db6	sym6	sym8
*SNR*	16.236	20.883	20.969	20.977	21.033
*MSE*	0.051	0.0174	0.01708	0.01706	0.0168
*RMSE_S_*	0.225	0.132	0.1307	0.1306	0.1298

**Table 5:** Corresponding frequencies of maximum amplitudes of signals under different powers.

No.	1	2	3	4	5	6	7	8	9	10	11
Laser power	0	50	100	150	175	185	195	205	215	225	230
Maximum amplitude (×10^−2^)	12.25	5.50	10.47	2.75	3.26	5.1	4.02	5.12	3.95	3.12	4.11
Frequency (kHz)	513.8	214.4	168.7	128.4	218.1	180.5	195.1	205.4	180.8	368.4	208.6

**Table 6:** Amplitude of frequencies near 515 kHz.

No.	1	2	3	4	5	6	7	8	9	10	11
Laser power	0	50	100	150	175	185	195	205	215	225	230
Frequency (kHz)	513.8	514.7	512.2	515.5	516.4	514.1	514.9	514.9	515.7	515.1	514.8
Amplitude (×10^−2^)	12.25	0.76	2.80	0.89	1.47	0.20	0.04	0.01	0.03	0.09	0.26

**Table 7:** Frequency band energy ratio.

No.	Laser Power	Frequency Band Energy Ratio (%)							
		*e*_1_	*e*_2_	*e*_3_	*e*_4_	*e*_5_	*e*_6_	*e*_7_	*e*_8_
1	0	1.74	50.51	13.21	17.43	0.83	0.89	13.08	2.30
2	50	4.96	52.19	7.13	25.85	1.26	1.34	5.01	2.26
3	100	2.35	61.24	6.77	17.64	1.17	1.52	6.17	3.16
4	150	16.16	60.43	4.26	15.54	0.15	0.26	2.49	0.70
5	175	8.16	60.93	4.60	22.20	0.19	0.34	2.57	1.00
6	185	3.89	61.85	7.36	23.04	0.34	0.56	1.47	1.50
7	195	4.04	66.72	4.35	22.81	0.07	0.22	1.01	0.77
8	205	2.03	68.85	2.77	23.16	0.23	0.52	1.23	1.20
9	215	4.44	56.67	7.17	29.68	0.03	0.11	1.22	0.69
10	225	2.82	44.05	9.16	41.24	0.15	0.22	1.05	1.31
11	230	4.61	48.36	9.63	31.17	1.09	1.06	2.21	1.87

## Data Availability

The data supporting the findings of this study are obtainable from the corresponding author upon reasonable request.
